# A Conscientious Patient

**Published:** 1853-04

**Authors:** 


*A Conscientious Patient.*—It is stated in the *Courier des Etats Unis,* that a dentist of high reputation, residing on the Boulevard, at Paris, was surprised by having his door bell rung with great violence every day for some time at precisely the same hour. On going to the door each time his servant found no visitor, but instead thereof, a five franc piece placed upon the mat. This was repeated for several days in succession, but the manner in which the money was placed there was finally discovered by waiting behind the door for the mysterious ringer, who was no other than an unhappy sufferer who came there every day for the purpose of having an aching tooth extracted. But on arriving at the door, the pain immediately ceased, which he attributed to the sudden approach of the dentist, whom with fastidious honesty he thus repaid. The dentist, who was an equally honest man, had much difficulty in persuading his singular patient to accept money, which the latter thought a very moderate remuneration for the benefit he had derived from approaching his door.

Dentists sometimes have patients who are not over conscientious with regard to paying for services actually rendered, and it may be there are some who would not object to being visited by twenty such as the mysterious ringer at the door of the Paris dentist, every day, but examples of the latter, it must be confessed, are rather rare.

